# The Impact of Variable Wind Shear Coefficients on Risk Reduction of Wind Energy Projects

**DOI:** 10.1155/2016/5790464

**Published:** 2016-10-30

**Authors:** Kenneth W. Corscadden, Allan Thomson, Behrang Yoonesi, Josiah McNutt

**Affiliations:** Engineering Department, Dalhousie University, Faculty of Agriculture, Truro, NS, Canada B2N 5E3

## Abstract

Estimation of wind speed at proposed hub heights is typically achieved using a wind shear exponent or wind shear coefficient (WSC), variation in wind speed as a function of height. The WSC is subject to temporal variation at low and high frequencies, ranging from diurnal and seasonal variations to disturbance caused by weather patterns; however, in many cases, it is assumed that the WSC remains constant. This assumption creates significant error in resource assessment, increasing uncertainty in projects and potentially significantly impacting the ability to control gird connected wind generators. This paper contributes to the body of knowledge relating to the evaluation and assessment of wind speed, with particular emphasis on the development of techniques to improve the accuracy of estimated wind speed above measurement height. It presents an evaluation of the use of a variable wind shear coefficient methodology based on a distribution of wind shear coefficients which have been implemented in real time. The results indicate that a VWSC provides a more accurate estimate of wind at hub height, ranging from 41% to 4% reduction in root mean squared error (RMSE) between predicted and actual wind speeds when using a variable wind shear coefficient at heights ranging from 33% to 100% above the highest actual wind measurement.

## 1. Introduction

Wind has been a major contributor to renewable energy sources, with large wind, both onshore and offshore, dominating the energy mix in many countries [1]. This trend will continue with wind expected to generate 12% of global electricity by 2020 and 20% by 2030. Much of this development will be in large scale wind; however, small wind is becoming a major player for both grid-tied distributed power generation and off-grid generation [2]. Accurate assessment of the wind resource is crucial in order to secure funding for projects, with many funding agencies requiring wind measurements at two-thirds of the proposed hub height; however, with increasing turbine sizes this is becoming more difficult, even with 60 m meteorological towers. Suitable forecasting models are an essential component of the assessment of proposed wind projects and the subsequent control and integration into grid-tied systems. Forecasting models and control technologies have received significant attention, evidenced by recent comprehensive reviews; Foley et al. [3] identify the significant advances in forecasting methods, encompassing statistical and physical models over varying time horizons and Mahela and Shaik [4] and Jain et al. [5] provide an equally detailed and enlightened review of the control strategies and prediction methods used to integrate wind into transmission and distribution networks. The necessity to accurately forecast wind speed is well documented and is generally based upon the Weibull probability density function, which is estimated from time series wind data typically obtained from a meteorological tower over extended testing periods [6]. The Weibull probability density function is a two-parameter function which is used to produce a wind speed profile for a particular site. Two parameters of shape (*k*) which is dimensionless and scale (*c*) in m/s are sufficient to characterize the Weibull function and are estimated from time series wind data typically obtained from a meteorological tower over extended testing periods. Of several recent regional studies testing the Weibull parameters, Weisser [7] analyzed two years of meteorological data in Grenada, West Indies, demonstrating the value of long term wind data to account for seasonal wind speed variations and the need for capturing the variation of wind speeds over the course of a day to account for diurnal changes but further highlight that typically two years of wind speed data is insufficient. Similarly, both Zhou et al. [8] and Lun and Lam [9] analyzed wind speed data, 1-year and 30-year data sets, respectively, both highlighting the value of the Weibull distribution, the need for long term data sets, and the need for wind-related assessments and evaluations. Statistical analyses of estimating the Weibull distribution of wind speed time series data are analyzed using various methods such as maximum likelihood, modified maximum likelihood, least squares, chi square, regression, graphical method, and methods of moments [10–12], with Seguro and Lambert [13] suggesting “maximum likelihood” as the recommended method, while Genc et al. [11] report the least squares method as producing better results for large sample sizes, while chi square is reported as providing the best overall fit [6]. The resulting Weibull distribution is then used to provide an estimate of potential power generation capacity, the basis for economic evaluation of a wind energy project for a particular site. Meteorological towers provide characteristics of wind speed near the earth's surface and it is therefore necessary to extrapolate wind speed to higher levels in the planetary boundary layer, particularly with higher turbine hub heights. This is achieved using a wind shear exponent or wind shear coefficient (WSC), variation in wind speed as a function of height, with two mathematical models of power law (PL) and Logarithmic Law (LL) used for extrapolation [14]. PL extrapolation is the most commonly utilized method for predicting wind speeds at a higher height than what is measured and historically uses a default exponent of 1/7th (0.142); however, research indicates that this value is neither stable on a diurnal, weekly, or seasonal basis, nor accurate for all sites due to varying surface roughness factors, atmospheric influences, and measurement heights [15, 16]. Firtin et al. [17], in a review of available WRAP (Winds Resource Analysis Program) data, found that 91.9% of wind shear coefficients were above 0.14, a clear indication that a default WSC may in some cases result in under- or overestimation of wind speeds and subsequently turbine Actual Energy Output (AEO). These findings were further supported by Rehman et al. [18] and Schwartz and Elliot [19], who identified a more realistic range of WSC of between 0.15 and 0.25. With the recognized issues of using a default 1/7th PL exponent for extrapolation, researchers have sought to modify the standard power law methodology and other extrapolation methods to better predict wind speeds at higher heights; however, in many cases, a fixed WSC is used based on long term average time series wind data. Farrugia [20] reported that while PL and LL were generally accepted for extrapolation up to heights of 100 m, significant variation in WSC occurred based on the month and time of day, a result that was also reported in a substantial study conducted by Bailey [21]. Ray et al. [22] conclude that there is little difference between the performance of the PL and LL models but noted greater variation in WSC with more complex terrain. Other researchers have investigated fundamental factors that impact wind shear, which includes its impact on turbine structures [23] and composite turbine blades [24], atmospheric stability, upwind terrain, surface roughness, sky condition (which contributes to night time radiative cooling), temperature, air-pressure, and humidity, in daily, seasonal, and directional trends. A common assumption however, that the WSC remains constant, has been identified by a number of authors as a contributing factor to increasing uncertainty in wind speed extrapolation. Lubitz [25] investigated the level of uncertainty associated with the PL model, concluding that the mean absolute error of extrapolated wind speed increased with increasing height above the measured wind speed and Irwin [26] proposed that variations in the power law exponent were impacted mostly by surface roughness and atmospheric stability, a factor that has more impact closer to the earth's surface. Fox [27] used friction velocity instead of a fixed WSC and applied this to utility scale turbines based on heights up to 150 m, claiming greater accuracy in wind speed extrapolation. Mikhail [28] used an alternative method referred to as a modified power law expression, claiming better accuracy. The degree of uncertainty in wind speed extrapolation has a much greater influence on energy production estimates [29]. Firtin et al. [17] investigated the impact of wind shear coefficients on electrical energy generation suggesting up to 49.6% error in energy production estimates using a PL extrapolation, with a fixed WSC. Several researchers such as Altunkaynak et al. [30] and Gualtieri and Secci [31] have attempted to address the uncertainty of WSC using a distribution, particularly the Weibull probability distribution to incorporate the temporal variation in WSC, using tower data. Şen et al. [32] consider an additional approach and combine the Weibull probability distribution with perturbation theory (which includes the standard deviations and covariance of wind speed at different elevations) to produce an extended PL, again incorporating time variations. Ðurišić and Mikulović [33] utilized a method of least squares (LES) and varied the shear exponent on a time-varying basis as a method of improving upon the traditional PL methodology. This methodology removes the concept of surface roughness and takes in to account the significant variation in WSC found on a diurnal and seasonal basis. Smedman-Hogstrom and Hogstrom, [34] developed a modified PL empirical model that incorporates the surface roughness in to the WSC calculation and Panofsky and Dutton developed a modified PL semiempirical model that estimates WSC as a function of surface roughness and stability [31]. Haque et al. [35] propose a new strategy for using computing models to predict short term wind speed, a method that has potential for both shear prediction and control systems. Significant advances have been made in the prediction of wind speed and there is evidence that such methods are now beginning to be considered for wind shear calculations as shown by Sintra et al. [36]. The evaluation of wind speed and wind shear is also inherently linked to control systems as such information is a prerequisite in the development of predictive control methodologies. The multivariable temporal variations in wind shear could be addressed using control theory and represented as a multivariable disturbance model, which has been demonstrated in other industries to result in improved controller performance [37]. This along and hybrid forecasting are emerging research opportunities [38]. Remote sensing of wind speed data using SoDAR is reported to provide wind speed measurements that correlate with anemometer data [39]. It has been proposed by Jeannotte [40] that SoDAR technology may have some limitations when used in complex terrain; however, there is merit in the contribution that SoDAR can make in addressing uncertainty associated with wind shear estimation, especially when applied to noncomplex terrain. Several researchers including Altunkaynak et al. [30] and Farrugia [20] have attempted to address the uncertainty of WSC using a distribution, particularly the Weibull probability distribution, to incorporate the temporal variation in WSC, using tower data. Şen et al. [32] consider an additional approach and combine the Weibull probability distribution with perturbation theory (which includes the standard deviations and covariance of wind speed at different elevations) to produce and extended power law, again incorporating time variations. A number of authors [35, 38, 41] have used advanced models to improve the characterization of wind speed and wind power estimates including neurofuzzy inference systems [42] and Markov Chain Models [43]. Bilgili et al. [44] utilized ANN to predict mean monthly wind speed at a target site using local reference wind tower data with some success but concluded that there is a need to ensure that reference wind towers must have a reasonable correlation factor (0.59). Fadare [45], Lee et al. [46], and Carolin Mabel and Fernandez [47] have all applied ANN models to specific geographic areas, with the authors highlighting the success of the ANN models in achieving reasonable accuracy in predicting wind speeds and the subsequent power output of wind turbines. However, while the use of ANN as a valuable tool is not disputed, Li and Shi [48] state that due to the number of different ANN models available and developed there are currently a number of factors that will influence forecasting accuracy including model structures, learning rates, and variation in required inputs. These models provide estimation at a single height, typically hub height using time series analysis, linear, nonlinear, and Artificial Neural Network (ANN) models, and subsequent hybrids, but there is little evidence of the same methods being applied to wind shear estimation. The estimation of WSC using a single fixed variable is an oversimplified approach creating challenges and increasing uncertainty in power production estimates for wind power projects. One must question the impact of such oversimplification and is the motivation behind the research presented in this paper. WSC research is possible using remote sensing, in this case, SoDAR technology to evaluate the potential of using a wind shear distribution (WSD), instead of a fixed WSC. This paper contributes to the research of wind shear estimation by presenting the results of an applied regional project conducted in the province of Nova Scotia, Canada. This research demonstrates the improvement in accuracy of wind speed estimation achieved using a WSD, validated using power prediction estimates for a commercial turbine, based on 60 m wind data with proposed hub heights of 80 m, 100 m, and 120 m. The paper examines the reduction in uncertainty and error obtained by using a variable of WSC creating a distribution instead of a fixed WSC to evaluate the accuracy of wind speed predictions at heights above measurement height.

## 2. Materials and Methods

**Figure 1:**
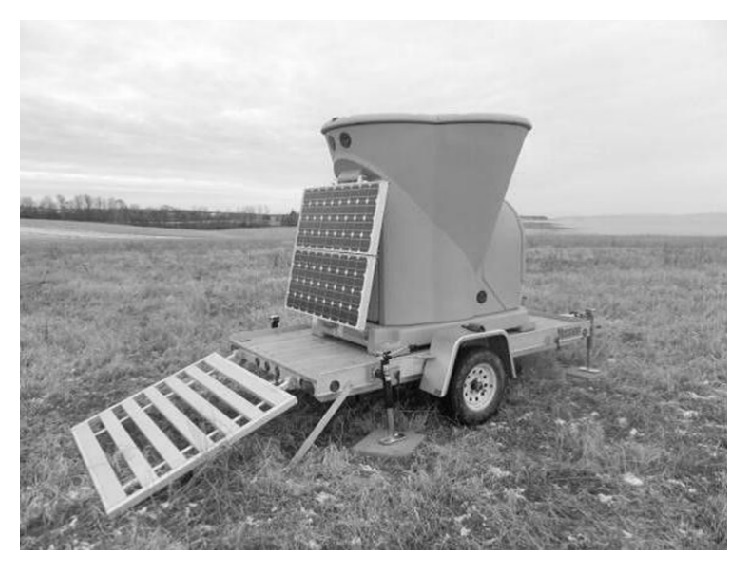
Trailer mounted SoDAR system.

Wind speed data was collected at eighteen different sites in Nova Scotia using a Vaisala Triton® Sonic Wind Profiler and SoDAR (Sonic Detection And Ranging), which uses the Doppler effect to reliably and accurately determine wind speed, wind direction, quality, and other operational parameters at heights ranging from 40 m to 200 m. The data collected by the SoDAR is sent via satellite to a “skyserve” website every 10 minutes, which is then downloaded to an excel file. The SoDAR, Figure 1, was mounted on a mission trailer for easy deployment and transportation between sites, the specification of which is listed in Table 1.

**Figure 2:**
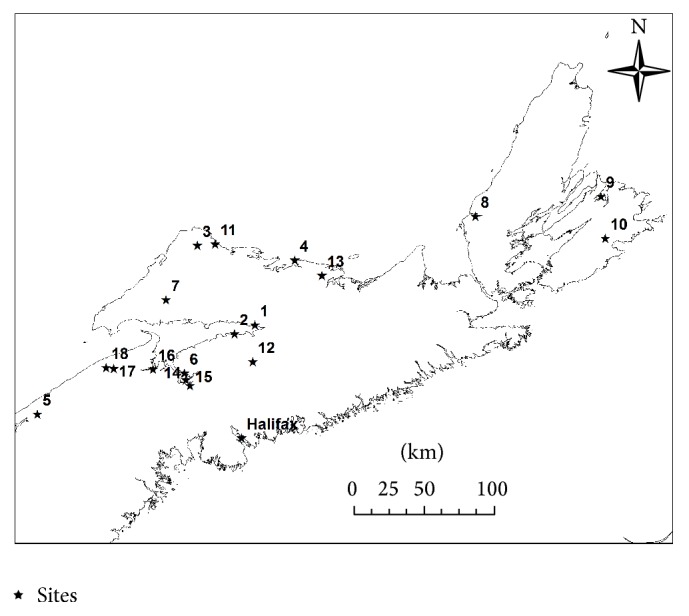
SoDAR deployment locations in Nova Scotia.

Deployment and commissioning of the SoDAR consist of selecting an appropriate position, leveling and securing the trailer. A Bushnell Scout 1000 range finder was used to ensure that the Triton was located a distance of at least 200 m from any obstruction, which includes buildings, trees, steep hills, or any crop that may be taller than the SoDAR, which may cause an echo and potentially corrupt the data. The SoDAR is oriented with the solar panels facing south to achieve maximum exposure to the sun. The Triton is then leveled within three degrees in all directions and secured using blocks. Propane tanks are installed inside the SoDAR which fuels a heater to avoid ice and snow buildup. In some cases, electric fences were installed to protect the SoDAR from livestock interference. The SoDAR was located at each of the 18 sites for at least three weeks. Site descriptors for the eighteen chosen locations are listed in Table 2 and displayed graphically in Figure 2. The raw SoDAR data for each site was imported to “Windographer” commercial wind analysis software for further investigation. The data for each site was analyzed to produce a Weibull distribution and subsequent average wind speeds for heights of 40 m, 50 m, 60 m, 80 m, 100 m, 120 m, 140 m, 160 m, 180 m, and 200 m.

According to the National Renewable Energy Lab classification, sites with wind power class three or higher are well qualified for large utility scale wind turbine applications, while sites with wind power class two (marginal) are not suitable for utility purpose but are suitable for rural applications [34]. Lastly, the sites with wind power class one are not suitable for utility scale or rural scale wind turbine application. Among the eighteen sites, fourteen sites had a wind power of class one with only four other sites identified as 2, 4, 11, and 13 having class two wind power. The objective of this research is to evaluate the impact of using a variable wind shear coefficient on the prediction of average wind speeds at heights above wind measurement height, estimate subsequent power production, and potential revenue stream of three turbines. The turbines selected include Enercon E48-800, EWT DW54-900, and Gamera G58-850, all of which have been selected because of the wind class.

## 3. Theory and Calculations

Two methods of wind speed prediction are used, both using the power law (PL) method for wind speed extrapolation, (1), to estimate the wind speed at 80 m, 100 m, and 120 m from a known reference height using a fixed wind shear coefficient (FWSC) and a variable wind shear coefficient (VWSC), where *V*_*i*_ represents wind speed at the new height *H*_*i*_, *V*_*io*_ is the speed at the original height *H*_*io*_, and “*α*_*i*_” is the wind shear coefficient for the* i*th site, where *i* = 4.

(1) $$\begin{array}{c} V_{i}=V_{io}\times {\left(\;\frac{H_{i}}{H_{io}}\;\right)}^{\alpha_{i}}. \end{array}$$

The two WSC prediction methods were evaluated by comparing the predicted wind speeds with the actual data collected using SoDAR at 80 m, 100 m, and 120 m for each site.

**Method 1 (fixed wind shear coefficient (FWSC)).**

Method 1 utilizes a fixed wind shear coefficient (FWSC). The FWSC is extrapolated using the average actual wind measurements obtained for the entire test period for each site, identified as *α*_(*i*)_, where subscript (*i*) represents the fixed wind shear coefficient for the* i*th site. The FWSC is then applied to the 60 m SoDAR wind data at hourly time steps to produce a synthesized data set at heights of 80 m, 100 m, and 120 m for each of the four sites. This requires a two-step process.

*Step  1*. For each of the four sites, the average actual wind speed data obtained using SoDAR at 40 m and 60 m, for the entire test period, was used to calculate a wind shear coefficient:

(2) $$\begin{array}{c} \alpha_{i}=\frac{\ln\left(V_{i,60}/V_{i,40}\right)}{\ln\left(H_{i,60}/H_{i,40}\right)}\;for\;\;i=1:4. \end{array}$$

*Step  2*. A synthesized wind data set, VF, was then produced, at three heights, for each of the four sites using (3), by applying the FWSC calculated for each site using (2) and the 60 m time series wind data set obtained using SoDAR with hourly time steps, where the* i*th subscript represents the site and the* j*th subscript each hourly time step in the synthesized time series data set.

(3) VFi,j,80=Vi,60×8060αifor  i=1:4VFi,j,100=Vi,60×10060αifor  i=1:4.VFi,j,120=Vi,60×12060αifor  i=1:4

**Method 2 (variable wind shear coefficient (VWSC)).**

The second prediction method utilizes variable wind shear coefficients (VWSC), creating a wind shear distribution. In this model, there are also two steps.

**Figure 3:**
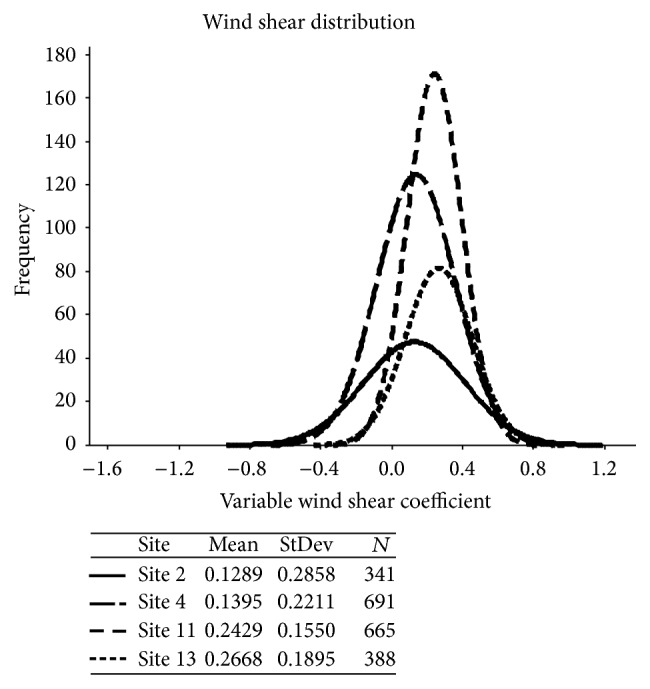
Variable wind shear coefficient distribution for each of the four sites.

*Step 1*. The average hourly wind speeds obtained at 40 m and 60 m for each site were used to create an hourly WSC, the combination of which creates a distribution, referred to throughout the remainder of the paper as a VWSC. The hourly time period was chosen to minimize the impact of high frequency wind speed variations and minimize noise in the prediction method. The daily VWSC obtained for each site is identified as *α*_(*i*,*j*)_, where the subscript (*i*, *j*) represents the* i*th site variable wind shear coefficient and* j* represents the hourly time step:

(4) αi,j=ln⁡Vi,j,60/Vi,j,40ln⁡Hi,j,60/Hi,j,40for  i=1:4,  k=1:y  over the test period,

where* y* is the number of hourly time steps obtained over the entire test period, resulting in the production of a distribution of wind shear coefficients for each site, Figure 3.

*Step 2*. The VWSC is then used to produce a corresponding estimate of wind speed Vv at 80 m, 100 m, and 120 m for each site:

(5) Vvi,j,80=Vi,j,60×8060αi,jfor  i=1:4Vvi,j,100=Vi,j,60×10060αi,jfor  i=1:4Vvi,j,120=Vi,j,60×12060αi,jfor  i=1:4.

These two methods therefore result in the production of six synthesized data sets for each of the four sites, two at 80 m, 100 m, and 120 m, respectively, one produced at each height using an FWSC, and the second produced using a VWSC where the variable coefficient distribution has been calculated every hour and is displayed in Figure 3.

The root mean square error, (6), was used to compare the accuracy of the synthesized time series wind data produced at each of the three heights for each of the four sites using an FWSC and a VWSC with the actual time series wind data obtained at 80 m, 100 m, and 120 m using SoDAR.

(6) $$\begin{array}{c} \text{RMSE}=\sqrt{\left(\frac{\sum_{i=1}^{n}{\left(V_{i,\text{predicted}}-V_{i,\text{actual}}\right)}^{2}}{n}\right)}. \end{array}$$

## 4. Results

**Figure 4:**
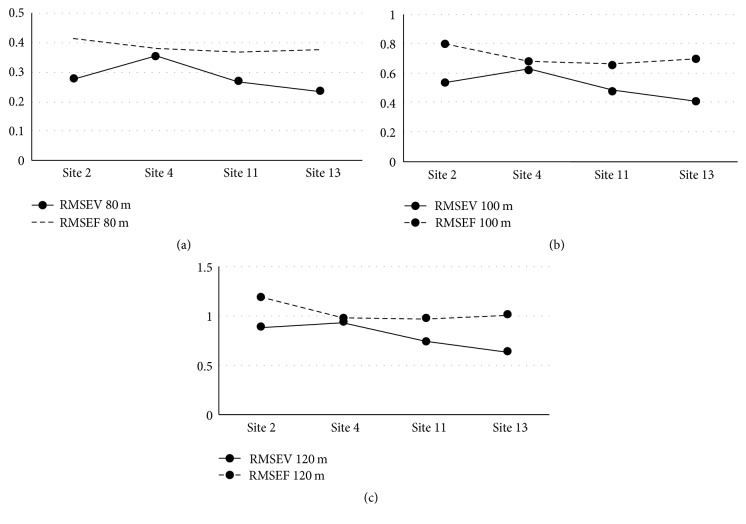
(a) 80 m wind speed RMSE. (b) 100 m wind speed RMSE. (c) 120 m wind speed RMSE.

The RMSE results for wind speed calculations at 80 m, 100 m, and 120 m for the class two sites for each of the two prediction methods are displayed in Table 3 and Figure 4. These results present the difference in RMSE between the synthesized wind speeds produced using the two methods, FWSC and VWSC, (6). Utilizing a VWSC, based on hourly time steps, produces a reduced RMSE at all 4 sites, between predicted and actual wind speeds at heights of 80 m, 100 m, and 120 m.

Figures 4(a), 4(b), and 4(c) show that the RMSE obtained at 80 m, 100 m, and 120 m are consistently better using the VWSC than that obtained using the FWSC. The improvement ranges from 41% to 4% reduction in root mean squared error (RMSE) between predicted and actual wind speeds when using a variable wind shear coefficient at heights ranging from 33% to 100% above the highest actual wind measurement. It is proposed that a variable WSC, or distribution, better reflects the true changes in wind speed as a function of height and time.

The goal of accurate wind speed prediction is to reduce uncertainty with annual energy output calculations and subsequent economic analysis of potential sites. Synthesized wind speed data sets at 80 m, 100 m, and 120 m were created for each site using each of the two wind speed prediction methods and applied to the wind turbine power curves for each of the three turbines. These predicted power outputs were then compared to the power outputs calculated using the actual wind speeds measured at 80 m, 100 m, and 120 m using SoDAR and presented as RMSE obtained using (6).

**Figure 5:**
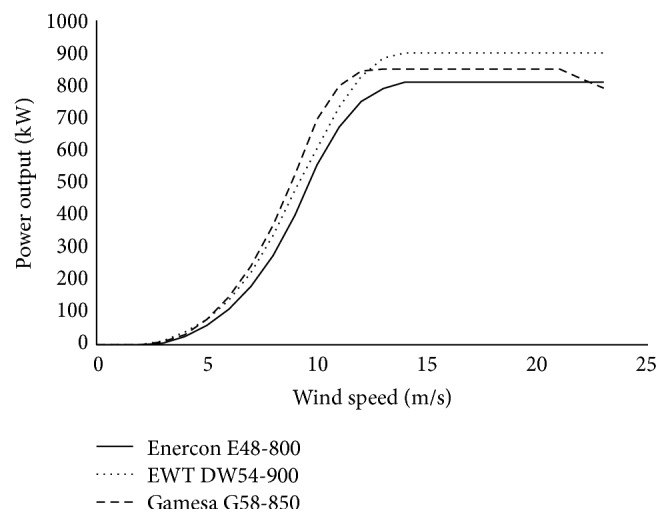
Wind turbine power curves for the three selected turbines.

The synthesized wind speeds produce synthetic data which then is applied to each of the turbine power curves to provide corresponding power calculations. The specifications and power curves of the wind turbine selected are listed in Table 4 and displayed in Figure 5, all of which have low start-up wind speeds of around 3 m/s and are available in North America.

### 4.1. Wind Turbine Power Output: Enercon E48-800

**Figure 6:**
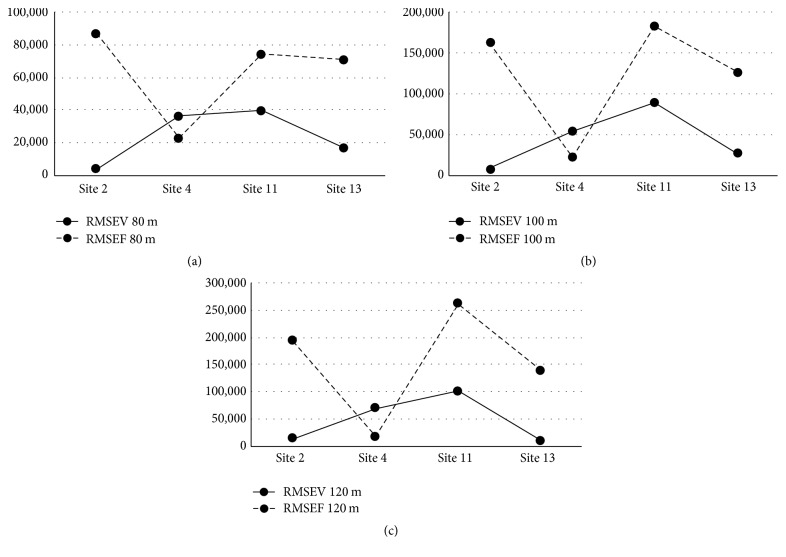
Enercon E48-800 AEP RMSE. (a) AEP RMSE, 80 m hub height. (b) AEP RMSE, 100 m hub height. (c) AEP RMSE, 120 m hub height.

The wind turbine power output for the Enercon E48-800 turbine for actual, fixed, and variable wind speed at 80 m, 100 m, and 120 m heights for each of the four sites is shown in Table 5. The RMSE results for wind power output calculations at 80 m, 100 m, and 120 m for each of class two sites for each of the two prediction methods are displayed in Table 6 and illustrated in Figures 6(a), 6(b), and 6(c).

### 4.2. Wind Turbine Power Output: Gamesa G58-850

**Figure 7:**
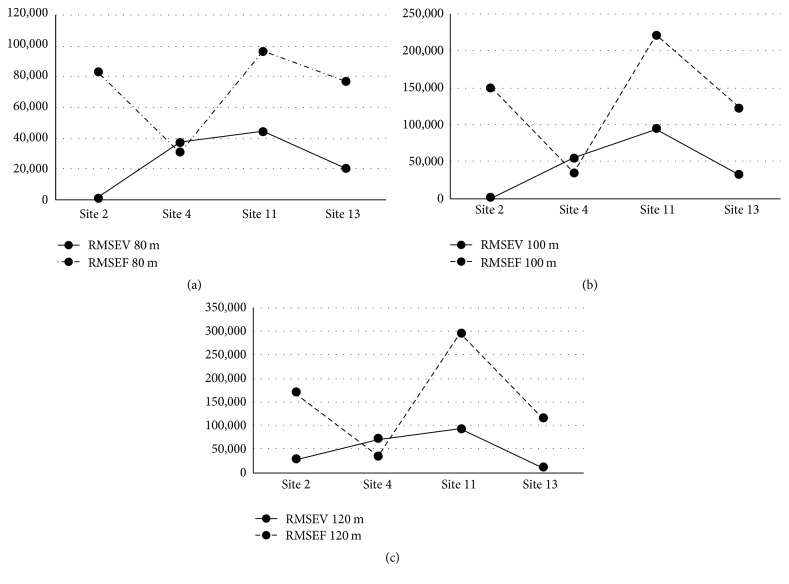
Gamesa G58-850 AEP RMSE. (a) AEP RMSE, 80 m hub height. (b) AEP RMSE, 100 m hub height. (c) AEP RMSE, 120 m hub height.

The wind turbine power output for the Gamesa G58-850 turbine for actual, fixed, and variable wind speed at 80 m, 100 m, and 120 m heights for each of the four sites is shown in Table 7. The RMSE results for wind power output calculations at 80 m, 100 m, and 120 m for each of class two sites for each of the two prediction methods are displayed in Table 8 and illustrated in Figures 7(a), 7(b), and 7(c).

### 4.3. Wind Turbine Power Output: EWT DW54-900

**Figure 8:**
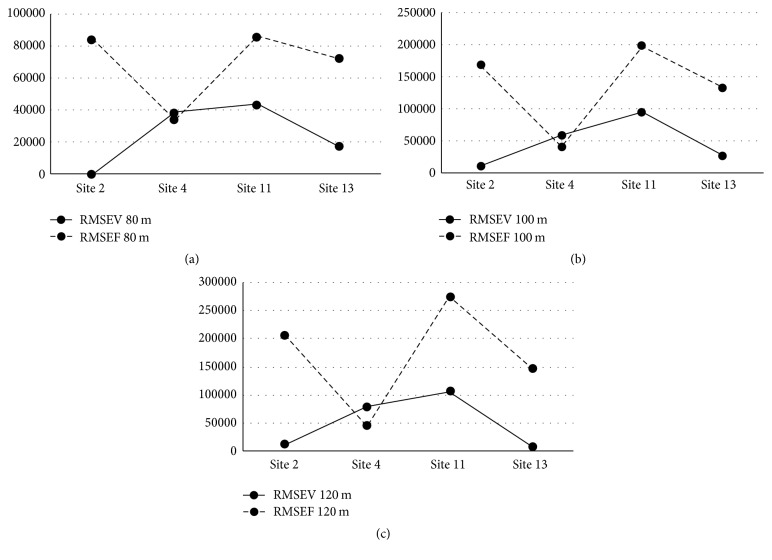
EWT DW54-900 AEP RMSE. (a) AEP RMSE, 80 m hub height. (b) AEP RMSE, 100 m hub height. (c) AEP RMSE, 120 m hub height.

The wind turbine power output for the EWT DW54-900 turbine for actual, fixed, and variable wind speed at 80 m, 100 m, and 120 m heights for each of the four sites is shown in Table 9. The RMSE results for wind power output calculations at 80 m, 100 m, and 120 m for each of class two sites for each of the two prediction methods are displayed in Table 10 and illustrated in Figures 8(a), 8(b), and 8(c).

### 4.4. Economic Analysis of Wind Production

The Province of Nova Scotia implemented a Community Feed in Tariff (COMFIT) [29] which paid $0.131 per kWh generated by wind turbines; however, this program has not ended. The Nova Scotia Government, as part of the Provincial Renewable Energy Plan, opened up the energy market to Independent Power Producers offering $90–$100/MWh for utility scale wind farms; however, this would not apply to a stand-alone wind turbine, and therefore the calculated Predicted Annual Revenue $/kWh is maintained at $13.1¢, as per the COMFIT program. By applying this rate to the wind turbine power generated for each of the two different methods for heights 80 m, 100 m, and 120 m, the predicted revenue generated for each turbine is presented in Tables 11 [Table tab12]–13.

By comparing these values with the predicted revenue calculated using the power generation obtained using actual wind speeds at 80 m, 100 m, and 120 m, (7), the percentage of error and hence the risk associated with the two different methods can be determined, the results of which are shown in Tables 14 [Table tab15]–16.

(7) $$\begin{array}{c} \%\text{ error}=\frac{V_{predicted}-V_{\text{actual}}}{V_{\text{actual}}}. \end{array}$$

The results presented in in Tables 14 [Table tab15]–16 provide some insight into the potential reduction of risk when using a distribution of wind shear coefficients and hence allowing a more accurate estimation of wind speed at heights above measurement height. This approach accounts for temporal variations and as such in almost all cases reduced the risk of projects. At sites 2, 11, and 13, the VWSC has a financial % error that is significantly lower than that obtained using the fixed method. At site 2, the FWSC method overestimates the revenue production by 5 to 10% while the VWSC overpredicts revenue by less than 1% error at all three heights. Site 11 has similar results ranging from 3% to 10% overprediction for the FWSC and only 2% to 4% overprediction when using the VWSC. Sites 13 has an overprediction range of 4% to 6% for the FWSC with 0.5% to 1.2% for the VWSC. Site 4 is the only one where the VWSC has a higher error than the FWSC; however, the error is positive meaning that the revenue production is underestimated.

## 5. Conclusion

This paper has presented a study based on measured wind data obtained at four different sites using SoDAR technology. The paper has compared the accuracy of wind speed predictions with actual wind measurements obtained using two methods of calculating wind shear coefficients. The results indicate that there is potential for significant reduction in RMSE and hence increased accuracy of wind speed prediction when using a distribution of wind speed coefficients with data at a range of heights above measured height. The results also show how such inaccuracies impact power prediction outputs with increasing height. The results suggest that encompassing a VWSC could significantly reduce uncertainty associated with wind speed estimation, power prediction, and revenue generation associated with wind energy project assessment. The next step in this research is to incorporate VWSC into predictive models to further enhance the accuracy of site assessment.

## Figures and Tables

**Table 1:** SoDAR Specifications.

Wind data capture range	40–200 m
Wind speed capture range	0–25 m/s
Filtered data correlation	Within 2% of anemometers
Nominal filtered data recovery rate at 100 m	90%–95% or higher

**Table 2:** Site description.

Site	Latitude	Longitude	Elevation (m)	Time elapsed (days)	Wind power class
1	45.37531	−63.45630	28	32	1
2	45.31687	−63.64144	31	34	2
3	45.88444	−63.98996	77	22	1
4	45.79366	−63.09253	26	36	2
5	44.77670	−65.41752	14	35	1
6	45.06085	−64.09318	45	36	1
7	45.53122	−64.27106	101	22	1
8	46.06527	−61.42274	20	23	1
9	46.17020	−60.25802	40	43	1
10	45.90073	−60.23143	86	23	1
11	45.89380	−63.82726	39	43	2
12	45.13878	−63.47387	95	21	1
13	45.69448	−62.84342	97	24	2
14	45.01728	−64.07950	45	14	1
15	44.98186	−64.04230	28	21	1
16	45.08299	−64.38023	44	44	1
17	45.08287	−64.73690	52	23	1
18	45.0877	−64.80604	230	42	1

**Table 3:** Wind speed RMSE for two prediction methods at 80 m, 100 m, and 120 m.

RMSE						
Site	RMSEV80	RMSEV100	RMSEV120	RMSEF80	RMSEF100	RMSEF120
2	0.277	0.5383	0.8802	0.4148	0.8022	1.1859
4	0.3549	0.6274	0.9334	0.3813	0.6824	0.9743
11	0.2692	0.4812	0.7357	0.3667	0.6624	0.9666
13	0.2353	0.4089	0.6291	0.3774	0.6988	1.005

**Table 4:** Wind turbine specifications.

Turbine model	Rated power	Wind class	Rotor diameter	Hub height (m)
Enercon E48-800	800 kW	IEC - IIA	48 m	50, 55, 60, 65, 76
EWT DW54-900	900 kW	IEC - IIIA	54 m	40, 50, 75
Gamera G58-850	850 kW	IEC -IIA/IIIB	58 m	44, 49, 55, 65, 74

**Table 5:** Wind turbine power output for Enercon E48-800.

Net AEP (kWh/yr) for Enercon E48-800									
Site	Variable			Fixed			Actual		
	80 m	100 m	120 m	80 m	100 m	120 m	80 m	100 m	120 m
2	1,640,339	1,834,187	1,992,187	1,557,492	1,680,627	1,784,254	1,644,432	1,843,249	1,977,211
4	1,833,872	1,984,803	2,114,853	1,820,445	1,953,188	2,063,269	1,797,344	1,930,490	2,045,135
11	2,170,278	2,465,171	2,731,790	2,136,041	2,372,395	2,572,013	2,210,066	2,554,526	2,833,121
13	1,969,887	2,280,955	2,535,078	1,916,173	2,182,055	2,406,725	1,986,843	2,308,628	2,545,774

**Table 6:** Wind turbine power output RMSE for Enercon E48-800.

Net AEP RMSE for Enercon E48-800						
Site	80 m		100 m		120 m	
	RMSEV	RMSEF	RMSEV	RMSEF	RMSEV	RMSEF
Site 2	4,093	86,940	9,062	162,622	14,976	192,957
Site 4	36,528	23,101	54,313	22,698	69,718	18,134
Site 11	39,788	74,025	89,355	182,131	101,331	261,108
Site 13	16,956	70,670	27,673	126,573	10,696	139,049

**Table 7:** Wind turbine power output for Gamesa G58-850.

Net AEP (kWh/yr) for Gamesa G58-850									
Site	Variable			Fixed			Actual		
	80 m	100 m	120 m	80 m	100 m	120 m	80 m	100 m	120 m
2	2,028,024	2,232,454	2,392,822	1,943,955	2,082,363	2,197,916	2,027,119	2,231,847	2,366,294
4	2,276,979	2,445,476	2,589,021	2,270,153	2,425,254	2,552,843	2,239,821	2,390,976	2,518,140
11	2,711,994	3,052,454	3,348,556	2,659,726	2,925,249	3,145,154	2,756,050	3,145,939	3,440,052
13	2,460,251	2,799,006	3,062,744	2,403,492	2,708,465	2,958,548	2,480,600	2,830,961	3,072,855

**Table 8:** Wind turbine power output RMSE for Gamesa G58-850.

Net AEP RMSE for Gamesa G58-850						
Site	80 m		100 m		120 m	
	RMSEV 80 m	RMSEF 80 m	RMSEV 100 m	RMSEF 100 m	RMSEV 120 m	RMSEF 120 m
Site 2	905	83,164	607	149,484	26,528	168,378
Site 4	37,158	30,332	54,500	34,278	70,881	34,703
Site 11	44,056	96,324	93,485	220,690	91,496	294,898
Site 13	20,349	77,108	31,955	122,496	10,111	114,307

**Table 9:** Wind turbine power output for EWT DW54-900.

Net AEP (kWh/yr) for EWT DW54-900									
Site	Variable			Fixed			Actual		
	80 m	100 m	120 m	80 m	100 m	120 m	80 m	100 m	120 m
Site 2	1,903,062	2,109,284	2,282,389	1,819,282	1,952,476	2,064,646	1,903,322	2,120,251	2,271,117
Site 4	2,126,366	2,293,489	2,437,733	2,121,769	2,275,454	2,403,107	2,087,667	2,235,122	2,359,198
Site 11	2,521,990	2,841,949	3,126,906	2,479,981	2,739,408	2,957,418	2,565,611	2,936,741	3,232,414
Site 13	2,289,489	2,627,862	2,907,482	2,234,621	2,522,097	2,768,711	2,306,779	2,654,928	2,915,576

**Table 10:** Wind turbine power output RMSE for EWT DW54-900.

RMSE for EWT DW54-900						
Site	80 m		100 m		120 m	
	RMSEV 80 m	RMSEF 80 m	RMSEV 100 m	RMSEF 100 m	RMSEV 120 m	RMSEF 120 m
Site 2	260	84040	10967	167775	11272	206471
Site 4	38699	34102	58367	40332	78535	43909
Site 11	43621	85630	94792	197333	105508	274996
Site 13	17290	72158	27066	132831	8094	146865

**Table 11:** Predicted annual revenue for Enercon E48-800.

Predicted annual revenue for Enercon E48-800									
Site	Variable			Fixed			Actual		
	80 m	100 m	120 m	80 m	100 m	120 m	80 m	100 m	120 m
Site 2	$214,884	$240,278	$260,976	$204,031	$220,162	$233,737	$215,421	$241,466	$259,015
Site 4	$240,237	$260,009	$277,046	$238,478	$255,868	$270,288	$235,452	$252,894	$267,913
Site 11	$284,306	$322,937	$357,864	$279,821	$310,784	$336,934	$289,519	$334,643	$371,139
Site 13	$258,055	$298,805	$332,095	$251,019	$285,849	$315,281	$260,276	$302,430	$333,496

**Table 12 tab12:** Predicted annual revenue for Gamesa G58-850.

Predicted annual revenue for Gamesa G58-850									
Site	Variable			Fixed			Actual		
	80 m	100 m	120 m	80 m	100 m	120 m	80 m	100 m	120 m
Site 2	$265,671	$292,451	$313,460	$254,658	$272,790	$287,927	$265,553	$292,372	$309,985
Site 4	$298,284	$320,357	$339,162	$297,390	$317,708	$334,422	$293,417	$313,218	$329,876
Site 11	$355,271	$399,871	$438,661	$348,424	$383,208	$412,015	$361,043	$412,118	$450,647
Site 13	$322,293	$366,670	$401,219	$314,857	$354,809	$387,570	$324,959	$370,856	$402,544

**Table 13:** Predicted annual revenue for EWT DW54-900.

Predicted annual revenue for EWT DW54-900									
Site	Variable			Fixed			Actual		
	80 m	100 m	120 m	80 m	100 m	120 m	80 m	100 m	120 m
Site 2	$249,301	$276,316	$298,993	$238,326	$255,774	$270,469	$249,335	$277,753	$297,516
Site 4	$278,554	$300,447	$319,343	$277,952	$298,084	$314,807	$273,484	$292,801	$309,055
Site 11	$330,381	$372,295	$409,625	$324,878	$358,862	$387,422	$336,095	$384,713	$423,446
Site 13	$299,923	$344,250	$380,880	$292,735	$330,395	$362,701	$302,188	$347,796	$381,940

**Table 14:** Percentage error from actual revenue generated for the two different methods: Enercon E48-800.

% error from actual revenue generated for Enercon E48-800						
Site	Variable versus actual % error			Fixed versus actual % error		
	80 m	100 m	120 m	80 m	100 m	120 m
Site 2	−0.250	−0.494	0.752	−5.58	−9.676	−10.814
Site 4	1.992	2.736	3.297	1.27	1.162	0.879
Site 11	−1.833	−3.625	−3.709	−3.47	−7.677	−10.152
Site 13	−0.861	−1.213	−0.422	−3.69	−5.801	−5.778

**Table 15 tab15:** Percentage error from actual revenue generated for the two different methods: Gamesa G58-850.

% error from actual revenue generated for Gamesa G58-850						
Site	Variable versus actual % error			Fixed versus actual % error		
	80 m	100 m	120 m	80 m	100 m	120 m
Site 2	0.04	0.027	1.1	−4.103	−6.7	−7.1
Site 4	1.659	2.279	2.8	1.4	1.4	1.4
Site 11	−1.6	−3.0	−2.7	−3.5	−7.0	−8.6
Site 13	−0.8	−1.1	−0.3	−3.1	−4.3	−3.7

**Table 16:** Percentage error from actual revenue generated for the two different methods: EWT DW54-900.

% error from actual revenue generated for EWT DW54-900						
Site	Variable versus actual % error			Fixed versus actual % error		
	80 m	100 m	120 m	80 m	100 m	120 m
Site 2	−0.01	−0.52	0.50	−4.42	−7.91	−9.09
Site 4	1.85	2.61	3.33	1.63	1.80	1.86
Site 11	−1.70	−3.23	−3.26	−3.34	−6.72	−8.51
Site 13	−0.75	−1.02	−0.28	−3.13	−5.00	−5.04
